# Isolation and in silico characterization of novel esterase gene with β-lactamase fold isolated from metagenome of north western Himalayas

**DOI:** 10.1007/s13205-014-0254-5

**Published:** 2014-10-19

**Authors:** Avneet Kour Sudan, Jyoti Vakhlu

**Affiliations:** School of Biotechnology, University of Jammu, Jammu, 180006 India

**Keywords:** β-Lactamases, Docking, DFP, Esterases, Homology modelling, Multiple displacement amplification

## Abstract

An esterase-producing clone Aph2 was isolated from the Apharwat soil metagenomic library, a mountain peak in NW Himalayas. ORF 2 (Est Ac) of clone Aph2 corresponds to 271 aa protein and showed 26 % sequence similarity to carboxylesterase gene of *Synechococcus* sp. JA-2-3B. Est Ac contains nucleophilic Ser in S^68^-X-X-K^71^ motif of β-lactamases with Tyr Y^103^. The conserved sequences are common with family VIII carboxylesterase and class C β-lactamase sequences. Phylogenetic analysis revealed that Est Ac sequence is closely related to esterase than to β-lactamases. In silico 3D protein structure of Est Ac was generated using MODELLER software (9.10 version). Model was generated on the basis of carboxylesterase template (PDB:1CI8) of Est B (*Burkholderia gladioli*) and the stereochemical parameters of the model generated were satisfactory. Docking with diisopropyl-fluorophosphate confirmed catalytic activity of Ser^68^ present in S-X-X-K motif.

## Introduction

Genes with wide industrial applications have been isolated from animals, plants and microbes with the largest source being the microbes (Wiseman 1995). Microbial genes are isolated and engineered by cultivation-dependent or cultivation-independent approaches. Cultivation-independent, metagenomic approach is favourable for the isolation of novel genes, due to absence of bias which is introduced by selective cultivation, cloning and PCR (Fuhrman 2012; Daniel 2005). The list of novel genes isolated using metagenomics is long and to name a few antibiotics, oxidoreductases, nitrile converting enzymes, glycerol hydratases, proteinases, lipases, esterases, amylases and antibiotic resistance genes (Gillespie et al. 2002; Knietsch et al. 2003; Liebeton and Eck 2004; Schmeisser et al. 2007; Morrohoshi et al. 2011; Berlemont et al. 2013; Vidya et al. 2011; Mullany 2014). The global enzyme market is expected to reach USD 7,652 million by 2020, growing at a CAGR of 8.3 % from 2014 to 2020 (http://www.grandviewresearch.com). Lipolytic enzymes being the most important class of enzymes, isolated and exploited for various industrial purposes, covering ~5 % of the global enzyme market (Vakhlu and Kour 2006). Lipolytic enzymes are categorised into eight families (I–VIII) on the basis of catalytic triad with residues Ser-His-Asp (Glu) in which the Ser-Oγ acts as a nucleophile present in pentapeptide G-X-S-X-G or GDSL motif (Arpigny and Jaeger 1999). Carboxylesterases (EC 3.1.1) are a subtype of lipolytic enzymes which hydrolyse carboxyl ester molecules to release alcohol and carboxylic acid. It is family VIII of carboxylesterases that shows major similarity with class C β-lactamases, peptidases and penicillin-binding protein and their primary sequence contains highly conserved S-X-X-K motif (where X is any amino acid) located at N-terminus of primary structure rather than the conventional G-X-S-X-G (Mokoena et al. 2013). The Ser residue which is known to act as catalytic nucleophile for family VIII carboxylesterase activity is present in the S-X-X-K motif and most of the members of this family are reported to lack activity against standard β-lactam substrates (Wagner et al. 2002). Prokaryotic genes are reported to show convergent evolution, wherein homologous catalytic motif having similar functions which may be present in different classes of hydrolases. Both classes, similarly family VIII carboxylesterases and β-lactamases possess catalytic residues in S-X-X-K motif of β-lactamase which is as a result of convergent evolution (Gherardini et al. 2007). In the present study, the isolation of a novel esterase gene from soil metagenome of Apharwat mountain peak in NW Himalayas having catalytic motif of β-lactamases is being reported. The niche is anthropogenically isolated and temperature variation is from 18° in summers to −20 °C in winters. The gene pool of the microbes in the Himalayas could be rich source for the novel enzymes and its potential has not been explored so far.

## Materials and methods

### Collection of soil sample

The soil sample was collected from Apharwat mountain (4267.2 m) with latitude 34.209° and longitude 74.368° during May 2007. The soil from a 5-cm-deep hole was collected in aseptic plastic bags that were then placed in containers to ensure that the microbial load was not disturbed and retained its natural form. Hands, trowels, and ice axes were treated with 70 % ethanol immediately before use. The samples were transported to the laboratory in dry ice and finally stored at −20 °C (Foght et al. 2004).

### Construction of metagenomic library

In the present study, DNA isolated using Wechter protocol (2002) contained impurities that hampered the manipulation of DNA. To reduce the interfering impurities and to increase the quantity of DNA, whole metagenome was diluted 50-fold and then multiple displacement amplification (MDA) was performed using phi (φ)29 DNA polymerase as per the method standardised previously (Sudan and Vakhlu 2012). Subsequent cloning and transformation generated 10,000 clones (Sudan and Vakhlu 2012).

### Sequencing and sequence analysis using bioinformatic tools and prediction of signal peptide

The sequencing was performed following Sanger’s dideoxy termination method at CIF South campus, Delhi University, India. Bioinformatic tools ORF Finder, BLASTP (http://www.ncbi.nlm.nih.gov.Blast) were used for functional ORF detection and sequence alignment, respectively.

### Multiple sequence alignment and phylogenetic analysis

Multiple sequence alignment was done using CLUSTALX (Larkin et al. 2007). The sequences selected for multiple alignment were retrieved from NCBI. Class C β-lactamase included that from *Candidatus kori* (Yp_589716), *Caulobacter* sp.K31 (Yp_001682441) and family VIII carboxylesterase of that of *Pseudomonas* sp. (gi AAA25813.1), esterase A *Streptomyces anulatus* (gi CAA78842.1) and Est B *Burkholderia gladioli* (PDB:1CI8) sequences. Evolutionary relationship between Est Ac, family VIII carboxylesterase and class C β-lactamase was inferred using neighbour joining method conducted with CLc software. Functional ORF was submitted to GenBank under accession number JX068525.1.

### Detection of signal peptide

Potential signal peptide in present case was retrieved using SIGNALP 3.0 server (Bendtsen et al. 2004).

### Comparative modelling of putative protein

Comparative modelling for protein 3D model generation was carried out by MODELLER 9.10 version software (Eswar et al. 2006). Est B was selected as template. Sequence of Est B was retrieved from PDB database. The modelling was based on alignment between sequences of Est B and Est Ac. The generated model was subjected to validation.

### Validation of model

Validation of the model generated was carried out using SAVs server including PROCHECK (Laskoswski et al. 1993) and ProSA (Wiederstein and Sippl 2007). These softwares were used to calculate the stereochemical evaluations.

### Ligand preparation and molecular docking

For in silico docking first the catalytic pocket was detected by CASTp software (Binkowski et al. 2003). The 3D descriptor (in SDF format) of diisopropyl-fluorophosphate (DFP) was retrieved from pubchem database (http://pubchem.ncbi.nlm.nih.gov). For in silico docking with diisopropyl-fluorophosphate (Wagner et al. 2002), the best orienting binding pose of ligand into the active site of the protein was estimated by using GOLD software in terms of its fitness score (Verdonk et al. 2003). The best fit pose generated a dock score which should be more than 30 (default). And ligand protein interaction, was indicated as hydrogen bonding and lipophilic interaction with other residues which was detected by LIGPLOT software (Wallace et al. 1995).

## Results and discussion

**Fig. 1:**
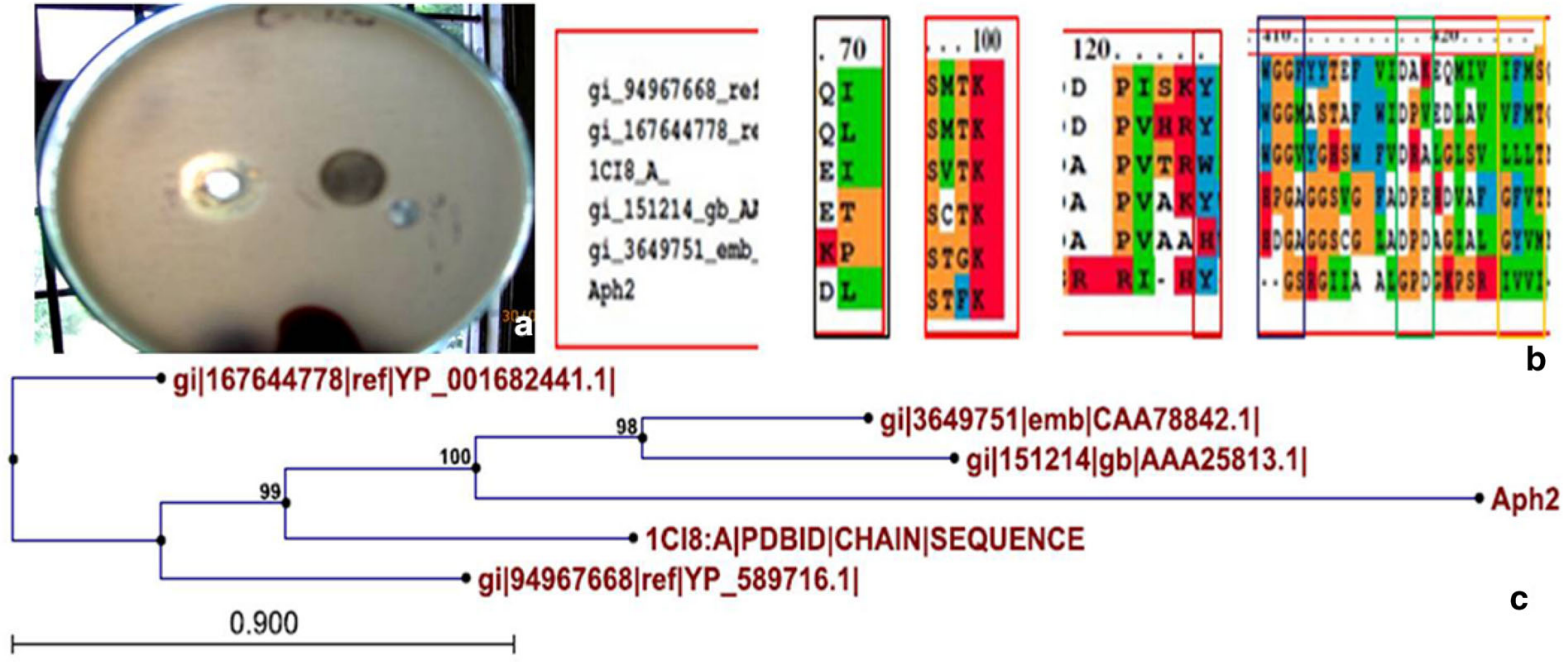
**a** Clone Aph2 harbouring Est Ac showing zone of hydrolysis on tributyrin plates. **b** Multiple sequence alignment using CLUSTAL X software between Est Ac (present study), with related members of family VIII carboxylesterases and class C β-lactamases were performed. The sequences used were retrieved from NCBI and were class C β-lactamase *Candidatus kori* (Yp_589716), *Caulobacter* sp. K31 (Yp_001682441), family VIII carboxylesterase were esterase *Pseudomonas* sp. (gi AAA25813.1) and esterase A *Streptomyces anulatis* (gi CAA78842.1) and Est B *Burkholderia gladioli* (PDB code 1CI8). The conserved amino acids are shaded and represented as Ser, Lys and Tyr that are positioned at S^68^-X-X-K^71^ and Y^103^, respectively, indicated in the *red boxes*. **c** Phylogenetic analysis of Est Ac and closely related class C β-lactamases and family VIII carboxylesterase sequences were generated using CLc software

Metagenomic library of soil metagenome isolated from Apharwat mountain peak in the north western Himalayas was constructed and 10,000 clones were screened for esterase activity on the tributyrin plates. Three clones showing esterase activity namely Aph2, Aph4 and Aph7 were isolated and preserved. An esterase-producing clone Aph4 has already been characterised (Sudan and Vakhlu 2012). In the present study, characterization of another esterase-producing clone Aph2 is being reported (Fig. 1a).

The insert in clone Aph2 harbours two non-overlapping functional ORFs as revealed by ORF Finder tool provided by NCBI (National Centre for Biotechnology Information) and the size of ORF 1 and ORF 2 of Aph2 was 356 and 811 bp, respectively. The nucleotide sequence of ORF 1 was 71 % similar to Endopeptidase lactopectin gene (*Bacillus* sp.), whereas ORF 2 (Est Ac) showed 26 % similarity with carboxylesterase gene (*Synechococcus* sp.) on BLASTP analysis (Table 1). Est Ac on analysis with NCBI tool (ORF Finder) suggested that it to encode for 271 aa enzyme that possess S-X-X-K motif of β-lactamases, wherein active catalytic Ser was conserved. Multiple sequence alignment of sequences of family VIII esterases, class C β-lactamases with Est Ac was carried out using CLUSTALX software. The sequences for multiple analysis were retrieved from NCBI (mentioned in “Materials and methods”, “Multiple sequence alignment” section). On multiple sequence alignment, the signature motif S-X-X-K and proton donor (Tyr) were found to be conserved in ORF 2 of clone Aph2. Ser present was found to be at position S^68^-X-X-K^71^ that acts as catalytic nucleophile characteristic of family VIII carboxylesterase. The nonconserved residues, however, in conserved motif of Est Ac were Thr and Phe making the signature sequence of this esterase, S^68^-T-F-K^71^ (Fig. 1b). Similar residues were found to be present in other cases like that of esterase Est C from *Burkholderia gladioli* (Wagner et al. 2002), lipase (LipBL) from halophilic bacterium *Marinobacter lipolyticus* SM19 (Perez et al. 2011), esterase Est M-N1 and Est M-N2 isolated from metagenomic DNA of Arctic soil sample (Yu et al. 2011), esterase Est C isolated from leachate fosmid shotgun library (Mokoena et al. 2013). However, the G-X-S-X-G motif of carboxylesterases and lipases that is known to be present along with S-X-X-K is absent in the present sequence. The esterase activity is dependent on S-X-X-K motif and G-X-S-X-G motif of lipases is reported to be non-functional (Petersen et al. 2001; Wagner et al. 2002; Perez et al. 2011; Yu et al. 2011; Mokoena et al. 2013).

**Table 1:** ORF description of Aph2 gene

S. no.	ORF name	Length	Frame	Accession no.	Similarity with	*E* value	Percentage of similarity
1	ORF 1 (118 a.a)	563–919 bp	−2	ZP_07104368.1	Endopeptidase lactopectin (*Bacillus* sp.)	2e−22	71
2	ORF 2 (271 a.a)	130–2,116	+3	YP_478112.1	Carboxylesterase with S-X-X-K motif of esterase (*Synechococcus* sp.)	4e−07	26

A phylogenetic tree was constructed using CLc Sequence software, wherein Est Ac is grouped into family VIII carboxylesterase instead of β-lactamases (Fig. 1c) as Est Ac clustered with reported sequences belonging to this family. The distance of Est Ac with selected family VIII carboxylesterase sequences were found to be remarkable, indicating Est Ac to be a novel protein.

### In silico modelling and signal peptide & catalytic centre detection

The nucleotide sequence was translated into amino acid sequence in silico using ORF Finder software. Est Ac was predicted to have 23 aa long N-terminus signal peptide using SIGNALP 3.0 server (Bendtsen et al. 2004). This could be cleaved to form mature protein (with a maximum cleavage site probability of 0.7 between Ala23 and His24). This could be a transmembrane protein as reported in similar study by Rashamuse and co-worker wherein 398 aa esterase Est C was obtained after the cleavage of 29 aa signal peptide from a protein of 423 aa (Rashamuse et al. 2009, 2011).

### Homology modelling of Est Ac using MODELLER software

**Fig. 2:**
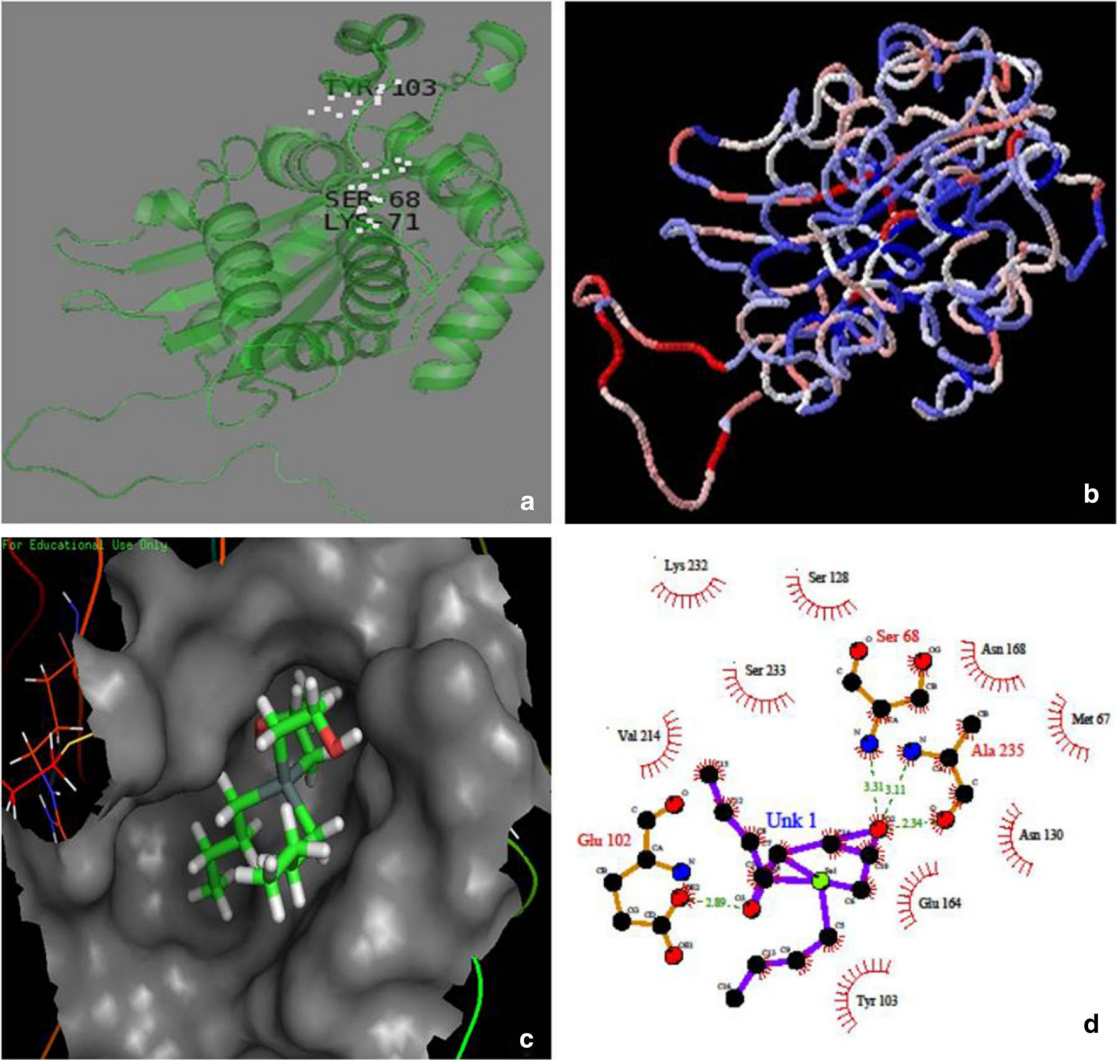
**a** Model generated for Est Ac using MODELLER (9.10 version) software. **b** For validation, CPH model generated by ProSA software was shown. **c** Molecular docking of Est Ac with diisopropyl-fluorophosphate (DFP) which was used for the detection of catalytic residue using GOLD software. **d** Detection of hydrogen bonds and hydrophilic interaction among the residues present in the Est Ac protein were visualised using LIGPLOT software

In addition to primary sequence analysis, structural insights were also generated by homology modelling using MODELLER (9.10 version) software (Eswar et al. 2006). Template IC18 (Est B isolated from *Burkholderia gladioli* that was confirmed by X-ray crystallography) was retrieved from PDB database and was used for model generation. Energies of models generated were calculated by the software in terms of their dope scores. In the present study, a total of five models were generated, model with least dope score, i.e. (−28911.082031) (Fig. 2a) was selected for future analysis. The model was visualised by using PyMOL software. Stereochemical parameters of the selected model was validated using SAVs SERVER and ProSA software (Laskoswski et al. 1993; Wiederstein and Sippl 2007) which were found to be satisfactory in the present case (Table 2). Stereochemical parameters included Ramachandran plot that showed no residues in the “disallowed regions” of ϕ/ψ space. The energy capacity was found to be accurate for the model which was calculated in terms of *z* score that determined overall quality which was −6.67. The local energy, however, determined by ProSA server that was also found to be negative. These results confirmed the model of good quality. The CPH model generated by ProSA software showed to contain all the residues in same place as that of the template thus validating the model (Fig. 2c). Wagner et al. (2002) constructed model for their protein Est B. The stereochemical parameters were found to be satisfactory. Ramachandran plot showed no residues in disallowed region of ϕ/ψ space with Ala^74^ and Asp^294^ being present in disallowed region.

**Table 2:** Stereochemical parameters studied using PROCHECK server which included Ramachandran plot, errat value calculation and *Z* score was calculated by ProSA software

Structural refinement and model	Est Ac (native)
Residue no.	271 aa
Bond length (%)	99.4
Bond angle (%)	94.1
Ramachandran plot (with procheck)	
Core region (%)	93.6
Generously allowed regions (%)	0.9
Not allowed regions (%)	0.0
G factors dihedral	0.07
Covalent	−0.10
Overall	0.00
M/C bond angle (%)	94.1
Overall quality factor (ERRAT2)	86.957
ProSA overall model quality (*Z* score)	−6.62

For carrying molecular docking a positive pocket possessing functional residues were determined using catalytic site atlas (CSA) software and CASTp software (Binkowski et al. 2003). In Est Ac catalytic residues Ser^68^ Lys^71^ Tyr^103^ were found in pocket number 37. It was found to be potent pocket for carrying docking with diisopropyl-fluorophosphate (DFP), a Ser inhibitor using GOLD software (Verdonk et al. 2003). It was observed that Ser^68^ found in S-X-X-K motif formed covalent interaction with DFP molecule through Oγ moiety (Fig. 2c). The GOLD score for the interaction determined was 48.0805. To study hydrogen bonding and lipophilic interactions between isolated protein and inhibitor (DFP), LIGPLOT software was used (Wallace et al. 1995). LIGPLOT generated hydrogen bonds of Ser^68^ along with other residues that may influence the catalytic power of the protein. The residues forming hydrogen bonds were Ala^235^ and Glu^102^ as is indicated with green dotted lines. However, the hydrophobic interaction were found to be seen in case of Lys^232^, Val^214^, Ser^128^, Asn^168^, Met^67^, Asn^130^, Glu^164^, Tyr^103^ (Fig. 2d). RMSD (root mean square deviation) difference of *C*_α_ atoms between the native structure and DFP derivative was calculated to be 0.20 Å. This interaction between Ser^68^ and ligand proved Ser^68^ to be potent binding catalytic residue for this inhibitor. Wagner and his group also reported the potent catalytic residue in their protein (Est B) which was found to be Ser^75^ present within β-lactamase Ser-X-X-Lys motif that acted as catalytic nucleophile and further suggested that covalent attachment of the inhibitor (diisopropyl-fluorophosphate) with their protein was made through Oγ of Ser^75^. RMSD difference of *C*α atoms between the native structure and the DFP derivative was 0.27 Å (Wagner et al. 2002) similar to the case reported in the present study. However, while conducting molecular docking it seemed that the acquisition of the inhibitor was from the top rather than from the front as reported by Wagner and his colleagues which further satisfied the claim of Est Ac to be an esterase and not a β-lactamase. The model generated falls well within the permitted limits suggesting it to be a valid model. The docking studies found Ser present in S-X-X-K motif as a potential nucleophile aiding in catalysis. Similar residues and similarities found in both Est Ac and Est B are given in Tables 3 and 4, respectively.

**Table 3:** Equalivalent residues of Est Ac and esterase (Est B)

Est B	Est Ac
Ser75	Ser68
Lys78	Lys71
Tyr133	Tyr103
Tyr181	Asn168
Val351	Val214

**Table 4:** Similarities between Est Ac (present enzyme) and Est B (Wagner’s enzyme)

S. no.	Similarity in properties	Est Ac (present enzyme)	Est B (Wagner’s enzyme)
1	Catalytic motif	S-X-X-K	S-X-X-K
2	Catalytic residues	S^68^-X-X-K^71^, Y^103^	S^75^-X-X-K^78^, Y^133^
3	KTG box	Absent	Absent
4	Docking molecule	DFP	DFP
5	Blocking residue	Ser^68^ through Oγ	Ser^75^ through Oγ

## Conclusion

The present study was conducted to explore the untreated Apharwat soil metagenome for its potential to isolate novel gene/s. A novel esterase gene was isolated which showed nucleotide sequence similarity largely with β-lactamases, but activity was seen on tributyrin plates. Sequence alignment studies and structural analysis proved that the isolated protein was a novel esterase with active catalytic residues Ser^68^ Lys^71^ Tyr^103^, also found in class C β-lactamase. This may be because of convergent evolution wherein the catalytic Ser is present in S-X-X-K motif (β-lactamase motif), common in case of β-lactamases and family VIII esterases.
